# The Relationships among Anxiety, Subjective Well-Being, Media Consumption, and Safety-Seeking Behaviors during the COVID-19 Epidemic

**DOI:** 10.3390/ijerph182413189

**Published:** 2021-12-14

**Authors:** Yi-Fang Luo, Heng-Yu Shen, Shu-Ching Yang, Liang-Ching Chen

**Affiliations:** 1 The Intelligent Electronic Commerce Research Center, Institute of Education, National Sun Yat-sen University, Kaohsiung 80424, Taiwan; a0989909301@gmail.com (Y.-F.L.); jokiceman@gmail.com (L.-C.C.); 2 Center for Teaching and Learning Development, National Kaohsiung University of Science and Technology, Kaohsiung 805301, Taiwan; 3 Department of Foreign Languages, R.O.C. Military Academy, Kaohsiung 83059, Taiwan

**Keywords:** COVID-19 epidemic, anxiety, media consumption, subjective well-being, safety-seeking behaviors

## Abstract

The COVID-19 epidemic has been confirmed as the largest scale outbreak of atypical pneumonia since the outbreak of severe acute respiratory syndrome (SARS) in 2003 and it has become a public health emergency of international concern. It exacerbated public confusion and anxiety, and the impact of COVID-19 on people needs to be better understood. Indeed, prior studies that conducted meta-analysis of longitudinal cohort research compared mental health before versus during the COVID-19 pandemic and proved that public health polices (e.g., city lockdowns, quarantines, avoiding gatherings, etc.) and COVID-19-related information that circulates on new media platforms directly affected citizen’s mental health and well-being. Hence, this research aims to explore Taiwanese people’s health status, anxiety, media sources for obtaining COVID-19 information, subjective well-being, and safety-seeking behavior during the COVID-19 epidemic and how they are associated. Online surveys were conducted through new media platforms, and 342 responses were included in the analysis. The research results indicate that the participants experienced different aspects of COVID-19 anxiety, including COVID-19 worry and perceived COVID-19 risk. Among the given media sources, the more participants searched for COVID-19 information on new media, the greater they worried about COVID-19. Furthermore, COVID-19 worry was positively related to safety-seeking behavior, while perceived COVID-19 risk was negatively related to subjective well-being. This paper concludes by offering some suggestions for future studies and pointing out limitations of the present study.

## 1. Introduction

In December 2019, a novel coronavirus disease (COVID-19) was discovered and started to spread worldwide [1]. The World Health Organization (WHO) [2] further declared the COVID-19 epidemic to be a public health emergency of international concern on January 30, 2020, which has caused high levels of public concern and fear about the possibility of a pandemic [1]. The media can provide fast and critical guidance regarding the pandemic [2]; however, different types of media may have different effects on coping. While traditional media (e.g., TV, newspapers, and radio) provide formal information about threats, new media (e.g., Internet and social media) has a more direct, personal impact on risk assessment [3]. New media may increase personal stress responses by sharing and viewing uncensored media content [4]. In addition, even new media may become a source of rapid dissemination of misinformation, aggravating public confusion and anxiety (Kim, 2019) [2] and thus negatively affect public health and well-being [5,6,7,8,9].

A meta-analysis of longitudinal cohort studies comparing mental health before versus during the COVID-19 pandemic in 2020 found an overall increase in mental health symptoms—e.g., [10,11,12,13,14]. Canet-Juric et al. (2020) assessed the citizen’s emotional impact of the lockdown measures implemented by the Argentinian government to fight the COVID-19 pandemic [15]. They surveyed the Argentinian general population twice (2 days after the mandatory quarantine started (time 1) vs. 2 weeks later (time 2). A total of 6057 people answered the two internet surveys and statistically significant variations were observed between the two time points. Their study suggested that it is necessary to continue monitoring mental health problems on the general population and necessary to create programs aimed at promoting mental health and to distribute information about it. Ramiz et al. (2021) conducted a longitudinal study of mental health, before and during the COVID-19 lockdown, in the French population [16]. They found, overall, people’s mental health deteriorated during the lockdown in France amid the 2020 COVID-19 pandemic. Moreover, their self-rated physical health improved but those who experienced a worse physical health were more likely to have mental health issues.

Anxiety is viewed as subsuming fear, panic, and worry, and it can be maladaptive, disrupting performance and interfering with both psychological and physical well-being [17]. Existing research results have shown that anxiety regarding the COVID-19 epidemic has a negative impact on health [18,19]. However, the literature also indicates that anxiety can trigger individual alertness and motivation to engage in safe behaviors that can promote survival and contribute to personal well-being [20,21]. The COVID-19 epidemic is a major public health event that involves the spread of the disease worldwide, and the impact of COVID-19 on people needs to be better understood [22].

Therefore, this research aims to understand Taiwanese people’s health status, anxiety about COVID-19, media sources for obtaining COVID-19 information, subjective well-being, and safety-seeking behavior during the COVID-19 epidemic.

### 1.1. Research Question and Hypothesis 1

The COVID-19 outbreak has caused public anxiety [6,22,23]. Anxiety, including complex emotional responses such as tension, fear, panic, and worry, is a very important concept in personality psychology [24]. Anxiety arises from the evaluation of a high degree of uncertainty about whether impending physical or psychological harm can be avoided [25]. Such an evaluation of uncertainty involves risk judgment, which includes perceived risk and worry [26]. Risk perception is a subjective cognitive assessment that involves the assessment of the probability of a specified negative accident occurring and the severity of consequences [27,28]. Worry is an emotional response, such as “feeling worried” when thinking about a risk source. According to the risk-as-feelings approach [29], cognitive assessment and worry have a reciprocal influence [26]. Therefore, this study intends to understand the anxiety state of participants during the COVID-19 epidemic and propose the following research questions:

*Q_1_: What is the participant’s COVID-19 anxiety, including the perceived risk and worry?*

For people with poor health, especially those suffering from certain diseases that have potential risks for infectious diseases due to the nature of the disease, catastrophic thinking about physical symptoms and overestimation of the risk of serious diseases may cause higher anxiety during pandemics [30,31]. The findings reported by Malesza and Kaczmarek (2021) also show that people with chronic diseases and poorer overall health have higher COVID-19 anxiety due to a greater perceived risk of infection [32]. Accordingly, we propose the following hypothesis:

**Hypothesis** **1** **(H1).** *Health status negatively predicts COVID-19 anxiety*.

### 1.2. Research Hypothesis 2 and Hypothesis 3

In public health crises, people believe that as much information as possible can help them understand the severity of the crisis which, in turn, helps them take protective action, reduce anxiety, and promote control over the situation [33]. In practice, however, public anxiety and stress for large-scale health crises may also be created by the media itself, the so-called media panic, which exists in different media sources, including newspapers, TV, radio, and the Internet. [34]. Although different types of media may have different effects on coping, little is known about the relationship between media source preference and audience response to large-scale pandemics [3].

Among many media sources, new media has become a research focus because new media platforms have been considered one of the most commonly used information resources [35]. Existing studies have shown that new media exposure may cause anxiety during a large-scale pandemic [16,36,37]. New media networks provide a new approach for combining and exchanging information [38], making it easy for Internet users, such as official departments, self-media, and netizens, to release and transfer related information on online media, which may lead to (mis)information overload and, in turn, cause individuals’ health problems, such as anxiety [6,33,37]. Compared with traditional media, the information quality of new media is out of control. Moreover, the interactive nature of new media is more likely to cause negative “emotional contagion” in disasters, which may cause new media users to experience more negative psychological effects [39]. Accordingly, we explore the relationship between different media sources and COVID-19 anxiety and propose the following hypotheses:

**Hypothesis** **2** **(H2).** *Higher anxiety to receive information from new media than traditional media*.

**Hypothesis** **3** **(H3).** *New media use frequency positively predicts COVID-19 anxiety*.

### 1.3. Research Hypothesis 3

Anxiety is associated with worse indicators of well-being [7]. Subjective well-being is a concept designed to evaluate the current life situation of an individual. Individuals with high subjective well-being give positive comments on their life conditions, while people with low subjective well-being give negative comments on their life conditions [8].

Existing studies have demonstrated that anxiety regarding COVID-19 affects individuals’ psychological well-being [5,9]. However, well-being is multidimensional [8]. Riediker and Koren (2004) [40] adopted the WHO (1948) definition of health, equating health with well-being [41] and explaining that well-being consists of physical, mental, and social elements. Existing studies mainly focus on the well-being of mental health but lack other dimensions of well-being. This research is expected to explore more comprehensive well-being and propose the following hypothesis:

**Hypothesis** **4** **(H4).** *COVID-19 anxiety is negatively related to subjective well-being*.

However, anxiety is also seen as an adaptive function that enables individuals to enhance their readiness for action when faced with ambiguous and unpredictable threats [20]. Therefore, proper anxiety about self-health helps individuals be alert to their own health and seek improvement [42]. In other words, anxiety is not only a result of health problems but also an alert and motivation that drives people to “seek safe behaviors” to effectively reduce threats [21]. Similarly, Li et al. (2020) noted that, in the face of potential disease threats, people tend to develop avoidance behaviors (e.g., avoid contact with people with pneumonia-like symptoms) and strictly follow social norms (e.g., conformity) [19]. Accordingly, we propose the following hypothesis:

**Hypothesis** **5** **(H5).** *COVID-19 anxiety is positively related to safety-seeking behaviors to prevent infection*.

**Figure 1:**
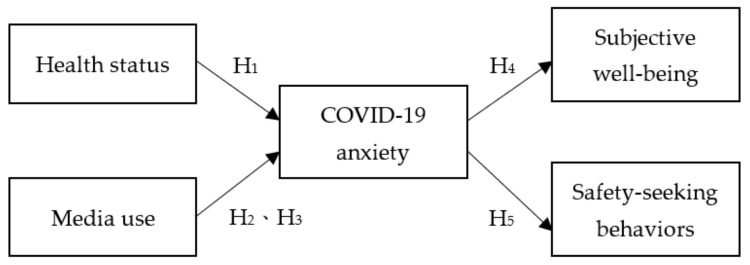
Research hypothesis framework.

The hypotheses that form the framework of this study are shown in Figure 1.

## 2. Methods

### 2.1. Studied Population

The study was conducted in 2020 and the research group consisted of 342 people. The characteristics of the study sample, including its sociodemographic characteristics, are presented in Table 1. The criteria for inclusion in the study were: age ≥ 18 years of age, Taiwan nationality, female or male gender. An anonymous online questionnaire was designed using a Google form in the traditional Chinese language that was accessible from any device with an Internet connection to invite potential respondents. The survey was disseminated via social networks (especially Facebook and Plurk) and respondents were encouraged to pass the survey on to others.

According to Taiwan’s “Ministry of Science and Technology (MOST) Communication Survey Database (four times in one phase) (2015): Political and Citizen Communication” (2002 interviewees in total), in terms of the frequency of receiving public affairs through traditional media, there is a significant difference between the men and women who are over 60 years old (*t* = 4.81, *p* < 0.05), while there is no significant difference between different sexes under 60 years of age. It can be found that in the younger generation, gender is no longer a factor that affects or limits the citizen’s reception of public affairs information. In addition, there is no significant difference in the frequency of using traditional media to receive public affairs among all interviewees of different age groups. In the section of new media, the difference is mainly the frequency of receiving public affairs between the younger and elder generations. Therefore, the population studied in this paper is mainly concentrated on students because they mainly use new media channels to finish the questionnaires [43].

### 2.2. Survey Instrument

#### 2.2.1. Health Status

Self-rated health status was measured by asking the participants how they felt in terms of their general state of health, and the responses ranged from “very poor” (1) to “very good” (5). This was one of the widely used validated indicators of health in the field of social sciences [44]. In this study, most of the participants rated their level of health as 4 (40.35%) or 5 (39.18%). In other words, the participants’ self-rated health tended to be good (*M =* 4.17, *SD =* 0.79).

#### 2.2.2. Media Consumption

The items were modified based on the media exposure measurement of Hong, Kim, and Xiong [45]. “Traditional media consumption” refers to the frequency of reading printed materials (such as newspapers and magazines), listening to the radio, and watching TV to obtain information related to COVID-19 (3 items). “New media consumption” refers to the frequency of obtaining COVID-19-related information from Internet news and social media. Responses were given on a 5-point Likert scale ranging from 1 (never) to 5 (always). The reliability and validity analysis showed that the factor loadings of “traditional media consumption” were, respectively, 0.88, 0.82, and 0.73, the total explained variance was 65.70%, and Cronbach’s α was 0.73. In “new media consumption”, the factor loads were 0.85 and 0.85, the total explained variance was 72.26%, and Cronbach’s α was 0.62.

#### 2.2.3. Subjective Well-Being

Subjective well-being mainly investigates the participants’ subjective perceptions of the impact of COVID-19 on their well-being. According to Riediker and Koren’s [40] definition of well-being, the study investigated the participants’ subjective well-being, namely physical health, mental health, and social relationships (including what do you think is the impact of COVID-19 on your physical health/mental health/social relationship?). The responses were given using a 9-point Likert scale ranging from −4 to 4, where a score of 0 indicates no impact at all, a score of −1 to −4 indicates a negative impact, and a score of 1 to 4 indicates a positive impact; thus, a more negative score indicates a greater negative impact of COVID-19 on well-being and vice versa. The reliability and validity analysis showed that the factor loadings ranged from 0.75 to 0.87, the total explained variance was 68.77%, and Cronbach’s α was 0.78.

#### 2.2.4. COVID-19 Anxiety

This study is based on the anxiety classification proposed by Rundmo and Nordfjærn (2017) [26] and references relevant literature (e.g., [42]) to compile this COVID-19 anxiety scale. The items were answered on a 5-point Likert scale ranging from 1 (strongly disagree) to 5 (strongly agree). The exploratory factor analysis (EFA) showed that the Kaiser–Meyer–Olkin (KMO) test value was 0.81 (χ^2^ =1416.79, *p* < 0.001) [46], the factor loadings ranged from 0.66 to 0.87, and the total explained variance was 69.75%. The scale was divided into two aspects: COVID-19 worry (e.g., worry individuals themselves or family will become infected with COVID-19, α = 0.88) and perceived COVID-19 risk (e.g., a high probability of becoming infected with COVID-19, very likely to be exposed to people with suspected or possible cases of COVID-19, α = 0.91).

#### 2.2.5. Safety-Seeking Behavior

Safety-seeking behavior assessed the participants’ degree of compliance with the government’s recommendations on preventing COVID-19 infection, including avoiding gatherings, maintaining social distance from others, maintaining hygiene habits of frequent hand washing, and wearing masks in indoor public places. The items were developed with reference to related literature—e.g., [1,22]. Responses were given on a 5-point Likert scale ranging from 1 (strongly disagree) to 5 (strongly agree). The reliability and validity analysis showed that the factor loadings were from 0.72 to 0.87, the total explained variance was 65.93%, and Cronbach’s α was 0.82.

### 2.3. Data Analysis

Statistical Product and Service Solutions 22.0 (SPSS) (IBM Corp, Armonk, NY, USA) software was used as a statistical tool in this study. A Kaiser–Meyer–Olkin (KMO) test was used to determine the sampling adequacy of data that were to be used for factor analysis [45]. The principal component analysis method with varimax rotation and eigenvalues >1 for EFA was adopted. Descriptive statistical analysis was performed to obtain a preliminary understanding of the respondents’ demographic characteristics and their health-related conditions, attitudes, behaviors, and literacy. A repeated-measures ANOVA or paired t-test and a simple or multiple regression analysis were used to analyze the data of this study.

### 2.4. Ethical Issues

This study followed the code of research ethics and conformed to the Taiwan government’s institutional review board rules for exempt review. We did not collect any relevant identifying information of the humans involved and an anonymous design questionnaire was used in this study. The questionnaire instructions clearly informed the participants of the research purpose and their rights regarding joining or dropping out of this study at any time during online filling-in. Participants were informed and assured that their participation was voluntary, anonymous, and strictly confidential and that they may stop participating in the study at any time without fear of penalty

## 3. Results

Table 2 provides a summary of descriptive analysis among demographic characteristics.

### 3.1. COVID-19 Anxiety

In response to the first research question, the study adopted a paired t-test, and the results showed that the participants’ worry about COVID-19 (*M =* 3.87, *SD =* 0.84) was significantly higher than the perceived risk of COVID-19 (*M =* 2.95, *SD =* 0.82) (*t*_(341)_ = 19.57, *p* < 0.001, Cohen’s *d* = 1.06).

### 3.2. New Media Consumption to Obtain COVID-19 Information

In response to the second research question, the study adopted a paired *t*-test, and the results showed that new media consumption (*M =* 4.37, *SD =* 0.62) was significantly higher than traditional media consumption (*M =* 2.35, *SD =* 0.85) (*t*_(341)_ = 37.19, *p* < 0.001, Cohen’s *d* = 2.72).

### 3.3. Analysis of Health Status and COVID-19 Anxiety

The results of the simple regression analysis showed that the participants’ health status significantly negatively predicted their perceived COVID-19 risk (beta = −0.24, *p* < 0.001, *R*^2^ = 0.06). However, there was no significant predictive relationship between health status and COVID-19 worry (beta = −0.02, *p* = 0.68). Thus, Research Hypothesis 1, that health status negatively predicts COVID-19 anxiety, was partially supported.

### 3.4. Analysis of Media Consumption and COVID-19 Anxiety

Table 3 shows that the frequency of new media consumption was significantly positively related to COVID-19 worry (beta = 0.23, *p* < 0.001) but not perceived COVID-19 risk (beta = 0.06, *p* = 0.28). In addition, the frequency of traditional media consumption was non-significantly positively related to COVID-19 worry (beta = 0.09, *p* = 0.09) and perceived COVID-19 risk (beta = 0.07, *p* = 0.22). Thus, Research Hypothesis 2, that new media use frequency positively predicts COVID-19 anxiety, was partially supported.

### 3.5. Analysis of COVID-19 Anxiety and Subjective Well-Being

Table 4 shows that the perceived COVID-19 risk was negatively related to physical health (beta = −0.16, *p <* 0.001) and mental health (beta = −0.21, *p <* 0.001). However, there were no significant predictive relationships between the two aspects of anxiety and social relationships. Thus, Hypothesis 3, which posits that COVID-19 anxiety is negatively related to subjective well-being, was partially supported.

### 3.6. Analysis of COVID-19 Anxiety and Safety-Seeking Behavior

Table 2 also shows that COVID-19 worry was significantly positively related to safety-seeking behavior (beta = 0.37, *p <* 0.001). However, perceived COVID-19 risk was not significantly related to safety-seeking behavior (beta = 0.01, *p* = 0.89). Thus, Research Hypothesis 4, that COVID-19 anxiety is positively related to safety-seeking behaviors to prevent infection, was partially supported.

## 4. Discussion

This study attempted to investigate Taiwanese people’s health status, anxiety, media consumption types, subjective well-being, and safety-seeking behavior during the COVID-19 epidemic. Consistent with previous findings, the study findings showed that new media was the most common source of information about COVID-19 [22]. As Internet and mobile communication technologies have been recently and widely integrated into our daily lives, online resources have become the main way for people to obtain information [47].

However, new media, which is a product of the development of the Internet, may exacerbate anxiety during the epidemic [23]. This study found that the participants experienced different aspects of COVID-19 anxiety and that these different aspects of anxiety had different relationships with media consumption, subjective well-being, and safety-seeking behavior. First, according to previous studies, anxiety arises from the evaluation of uncertainty [25] and includes perceived risk and worry [26]. The results of this study also revealed the complexity of anxiety during the COVID-19 epidemic. In this study, COVID-19-related anxiety was divided into the following two aspects: COVID-19 worry, including worry about the infection of oneself and one’s relatives and friends and worry about the outbreak and return of the epidemic; and perceived COVID-19 risk, including the perceived risk of the possibility of infection with COVID-19 and exposure to people with suspected cases and the perceived possible consequences of COVID-19 infection when going out, despite taking preventive measures.

Furthermore, this study found that, although the participants reported a low perceived risk of COVID-19, they had high levels of worry about COVID-19. Emotional responses to risky situations and cognitive assessments of those risks are often inconsistent [29]. Therefore, when faced with extremely undesirable outcomes, people will still have a high level of anxiety, despite the low probability of these outcomes [25]. In other words, when faced with extremely undesirable outcomes, the anxiety caused by the emotional response is more critical than the anxiety caused by the cognitive evaluation.

In addition, when an emotional response to risk diverges from a cognitive evaluation of risk, the emotional response is often the predominant predictor of risk-related behavior [25,29]. Consistent with previous studies, this study found that COVID-19 worry, but not perceived COVID-19 risk, was positively related to safety-seeking behavior. However, this study also found that the frequency of new media consumption was positively related to COVID-19 worry. The relationship between new media and emotional responses may be due to the viral spread of misinformation and false reports about COVID-19 in new media during the epidemic, which has caused unfounded fear among many netizens, with the potential to confuse people and cause anxiety (Kim, 2019) [2]. In addition, many netizens have expressed their negative emotions, such as fear, worry, tension, and anxiety, through new media, which, in turn, has caused negative emotional contagion in the online community [23].

Finally, this study found that the participants’ self-rated health status was poorer and their anxiety from perceived COVID-19 risk was higher. In an epidemic, it is common for individuals to feel stressed [48], which leads to anxiety [17]. In particular, people with poor health are more likely to experience anxiety from the stress of the epidemic [22]. According to Lundberg (1998) [49], the degree of stress depends on an individual’s cognitive evaluation of danger and potential injury. Therefore, people with poorer overall health tend to consider physical symptoms catastrophically and overestimate the risk of serious diseases, which may cause higher anxiety during pandemics [30,31,32]. The results also echoed with Robinson et al.’s meta-analysis of longitudinal cohort studies, revealing that when comparing mental health symptoms to pre-pandemic levels, larger rises for depressive symptoms and those with existing poor physical health may have been most affected [14].

The study found that risk was not significantly related to safety-seeking behaviors to prevent infection, only worry was significantly positively related to safety-seeking behavior. The researchers infer that it may be related to the temporal and spatial backgrounds of the pandemic. It was before the COVID-19 outbreak in Taiwan, and therefore citizen’s awareness of COVID-19 risk was relatively low. However, through media reports, people began to know the catastrophe that COVID-19 caused in other severely affected areas, and they may have started to worry about the impacts of the virus and whether it would infect themselves and their relatives and friends. This paper suggested that future research can further explore where there exist other intervening variables, for example, whether factors that the health status of participants may cause such differences.

Anxiety may further reduce well-being [7]; that is, anxiety may lead to worse physical and mental health [50]. Existing studies have demonstrated that anxiety regarding COVID-19 affects individuals’ psychological well-being [5,9]. This study has similar findings, finding that anxiety from perceived COVID-19 risk has a negative impact on the well-being of physical and mental health.

However, this study found that anxiety has no significant predictive relationship with the well-being of social relationships. This may be because, even though the Taiwanese government implemented some regulations to prevent the spread of COVID-19, including delaying the start of the new semester for schools, restrictions on the number of people at large indoor and outdoor gatherings, social distancing, and wearing masks, there were no stringent restrictions on movement and no local or national lockdown [51]. Furthermore, the development of the Internet makes being online provide opportunities to connect with families, friends, and other people from beyond communities [52]. Therefore, even if COVID-19 causes inconvenience in face-to-face interpersonal relationships, people can still seek online ways to maintain interpersonal relationships. The above reasons may cause people’s COVID-19 anxiety to have less impact on the well-being of interpersonal relationships.

## 5. Conclusions

This study revealed that new media has become the main source of COVID-19 information and the more participants searched for COVID-19 information on new media, the greater they were worried about COVID-19. Therefore, this study suggests that it is necessary to ensure the accuracy of COVID-19-related information that is communicated to the public. In particular, individuals with poor health are more likely to be vulnerable because of anxiety during the epidemic. Therefore, it is necessary to pay more attention to the anxiety of these vulnerable groups during the COVID-19 epidemic. In addition, this study revealed that COVID-19 worry is an emotional response rather than a cognitive assessment and that COVID-19 worry helps people engage in preventive behavior. However, whether anxiety caused by an excessive emotional response will cause undesirable behavior, such as unnecessary visits to emergency departments or the hoarding of face masks [6], needs further exploration.

Future studies may need to further consider participants’ demographic information (e.g., socioeconomic status, gender, age groups, occupation), relevant factors (e.g., physical health conditions, resilience, protective factors, psychological adjustment, coping strategy), and mixed methods (e.g., qualitative, longitudinal) in understanding the relationships among examined constructs, and to further examine the change over time and whether the changes are persistent or short lived, and if changes were symptom specific.

This study had some limitations. Although new media, such as search engines, social media apps, online discussion boards, etc., has changed the ways we retrieve and acquire information, fake news and false reports (information) occur frequently and make people panic or cause some mental diseases, especially amid the COVID-19 pandemic. With the advancement of information, communication, and technology (ICT), it is important to explore the impacts of the aforementioned issues. Hence, this paper mainly focused on investigating the citizen who mainly relies on new media channels to obtain information. The survey respondents are mostly young people, as this group of citizens may spend more time on smartphones or computers than other groups and have a high likelihood of accessing and finishing the online surveys of the present study, which is also consistent with the results of the Taiwan MOST Communication Survey Database (2015) [43]. The research results may not be analogized to other population groups (e.g., middle-aged, senior citizens, etc.). Nevertheless, this research only used the new media platforms as the primary survey channel because the researchers valued the social issues of the new media, but the derived problem is that the results may not be widely applicable to non-social media users. Thus, it is suggested that future research can investigate the anxiety, subjective well-being, media consumption, and safety-seeking behaviors amid the COVID-19 epidemic in different population groups through multiple ways.

Because of individual subjectivity, participants’ self-reports may not reflect their actual media consumption behavior and safety-seeking behavior. Furthermore, although in a statistical sense, health status and new media use frequency can predict COVID-19 anxiety, and COVID-19 anxiety can predict subjective well-being and safety-seeking behaviors, in a practical sense, these variables are related but not necessarily causally related. Therefore, other diversified research methods can be used in future research to clarify the relationship between these variables. Another limitation of this study was that subjective well-being investigated only physical health, mental health, and social relationships. However, subjective well-being is an individual’s evaluation of life conditions, and the life of human beings contains broader aspects. It is suggested that future research should continue to track the impacts of different aspects of COVID-19-related anxiety on broader aspects of life, such as the economy. Although this study had limitations, it is still helpful for understanding the relationship between anxiety and related variables during the COVID-19 epidemic and can be regarded as a basis for subsequent research development.

## Figures and Tables

**Table 1:** Participant demographic characteristics.

Variables	Category	*N*	%
Gender	Woman	146	42.69%
	Man	196	57.31%
Age	<20	140	40.94%
	21–30	174	50.88%
	>31	28	8.18%
Occupation	Student	284	83.04%
	Non-student	58	16.96%
Education	Associate’s degree	21	6.14%
	Bachelor’s degree	249	72.81%
	Master or doctoral degree	72	21.05%

**Table 2:** Summary of descriptive analysis among demographic characteristics.

Variables Category	COVID-19 Worry	Perceived COVID-19 Risk	Physical Health	Mental Health	Social Relationship	Safety-Seeking Behavior
**Gender**						
Woman	3.90 (0.88)	2.99 (0.84)	−0.40 (1.50)	−0.75 (1.54)	−0.07 (1.17)	4.17 (0.63)
Man	3.84 (0.80)	2.92 (0.80)	−0.50 (1.17)	−0.76 (1.12)	−0.31 (0.99)	4.21 (0.63)
**Age**						
<20	3.88 (0.86)	2.97 (0.90)	−0.39 (1.21)	−0.71 (1.21)	−0.12 (1.04)	4.11 (0.67)
21–30	3.90 (0.85)	2.95 (0.76)	−0.53 (1.40)	−0.84 (1.37)	−0.21 (1.04)	4.25 (0.60)
>31	3.58 (0.63)	2.79 (0.77)	−0.32 (1.33)	−0.43 (1.43)	−0.61 (1.40)	4.29 (0.54)
**Occupation**						
Student	3.88 (0.85)	2.94 (0.81)	−0.49 (1.30)	−0.80 (1.29)	−0.17 (1.00)	4.16 (0.64)
Non-student	3.81 (0.80)	2.98 (0.87)	−0.28 (1.41)	−0.53 (1.44)	−0.41 (1.38)	4.35 (0.53)
**Education**						
Associate’s	3.77 (0.83)	2.90 (0.68)	−0.10 (1.64)	−0.76(1.34)	−0.24 (0.77)	4.12 (0.73)
Bachelor’s	3.89 (0.81)	2.94 (0.84)	−0.51 (1.20)	−0.74 (1.27)	−0.19 (1.08)	4.21 (0.64)
Master’s and above	3.82 (0.93)	2.98 (0.81)	−0.39 (1.60)	−0.81 (1.47)	−0.26 (1.14)	4.17 (0.55)

**Table 3:** Multiple regression analysis of media consumption.

Variable Media Consumption	COVID-19 Worry		Perceived COVID-19 Risk	
	*Beta*	*t*	*Beta*	*t*
**Traditional media**	0.09	1.71	0.07	1.22
**New media**	0.23	4.26 ***	0.06	1.09
	*R* = 0.25 *R*^2^ = 0.06 *F*_(2339)_ = 11.17 ***		*R* = 0.09 *R*^2^ = 0.01 *F*_(2339)_ = 1.45	

*** *p <* 0.001. *Beta*: standardized coefficients.

**Table 4:** Multiple regression analysis of COVID-19 anxiety.

Aspect	Physical Health		Mental Health		Social Relationship		Safety-Seeking Behavior	
	*Beta*	*t*	*Beta*	*t*	*Beta*	*t*	*Beta*	*t*
**COVID-19 worry**	−0.06	−0.98	−0.07	−1.26	0.07	1.18	0.37	6.45 ***
**Perceived COVID-19 risk**	−0.16	−2.63 ***	−0.21	−3.48 ***	−0.04	−0.63	0.01	0.14
	*R* = 0.19		*R* = 0.25		*R* = 0.06		*R* = 0.37	
	*R*^2^ = 0.04		*R*^2^ = 0.06		*R*^2^ = 0.004		*R*^2^ = 0.14	
	*F*_(2339)_ = 6.42 **		*F*_(2339)_ = 11.10 ***		*F*_(2339)_ = 0.70		*F*_(2339)_ = 26.66 ***	

** *p <* 0.01. *** *p <* 0.001. *Beta*: standardized coefficients.

## Data Availability

The data presented in this study are available on request from the corresponding author.
